# Quantification of normetanephrine in canine urine using ELISA: evaluation of factors affecting results

**DOI:** 10.1177/10406387211052984

**Published:** 2021-10-26

**Authors:** Katja Höglund, Hanna Palmqvist, Sara Ringmark, Anna Svensson

**Affiliations:** Departments of Anatomy, Physiology and Biochemistry, Swedish University of Agricultural Sciences, Uppsala, Sweden; Animal Nutrition and Management, Swedish University of Agricultural Sciences, Uppsala, Sweden; Departments of Anatomy, Physiology and Biochemistry, Swedish University of Agricultural Sciences, Uppsala, Sweden; Clinical Sciences, Swedish University of Agricultural Sciences, Uppsala, Sweden

**Keywords:** acidification, catecholamine, dogs, metanephrine, metanephrines, normetanephrine, sample storage

## Abstract

Catecholamine release increases in dogs with pheochromocytomas and in situations of stress. Although plasma catecholamines degrade rapidly, their metabolites, normetanephrine (NME) and metanephrine (ME), are stable in acidified urine. Our aim was to verify a human urine ELISA kit for the quantification of NME and ME in canine urine and to determine the effects on metabolite stability of sampling time (morning or midday) and day (ordinary or day spent in a clinic). We analyzed 179 urine samples from 17 healthy dogs. For NME, the mean intra-assay CV was 6.0% for all samples and 4.3% for the canine control; inter-assay CVs were 3.3, 3.8, and 12% for high and low concentration human urine positive controls supplied in the ELISA kit and a positive canine control, respectively; spike-recovery was 90–101%. For ME, mean intra-assay CV was 6.5% for samples and 9.0% for the canine control; inter-assay CVs were 12.7, 7.2, and 22.5% for high and low concentration human urine positive controls supplied in the ELISA kit and a positive canine control, respectively; spike-recovery was 85–89%. Dilution recovery was unsatisfactory for both metabolites. Based on our verification results, NME was selected for remaining analyses. We found no effect on NME concentrations of acidification or room temperature storage for up to 24 h. The NME:creatinine ratio was higher after the first of 3 clinic days compared to the same morning (111.2 ± 5.5 vs. 82.9 ± 5.3; *p* < 0.0001), but not on the other days. NME verification results were generally superior to ME. Dilution studies were unsatisfactory for both metabolites. Given that NME was stable without acidification at room temperature, urine samples can be collected at home. The clinic environment can cause higher NME:creatinine ratios, especially in unaccustomed dogs.

Measurement of catecholamines and their metabolites is used for assessment of sympathetic stimulation and for diagnosis of neuroendocrine tumors, such as pheochromocytomas.^11,15,16,20,22,25^ In human plasma, the half-life of catecholamines is only a few minutes.^12^ Furthermore, because catecholamines are sensitive to oxidation in canine and human plasma samples, acidification of samples and rapid processing is needed to reduce degradation.^3,5,7^ Catecholamines are relatively stable in human urine samples, with the highest stability in acidified samples.^3,24^

Activation of the sympathetic nervous system leads to increased release of epinephrine and norepinephrine from the adrenal medulla, as well as release of norepinephrine from adrenergic nerve endings. Norepinephrine and epinephrine are degraded by catechol-O-methyltransferase to normetanephrine (NME) and metanephrine (ME), respectively.^8,20^ These metabolites, collectively called metanephrines, are stable and have been found in higher concentrations than catecholamines in canine urine, as well as in human plasma and urine.^10,15,16,19,30^ However, acidification of urine samples is still recommended by manufacturers of assays for the metabolites.

Handling and examination in a clinic environment can be stressful for dogs,^13,27^ thus potentially affecting analytical results. Sampling of voided urine collected at home may give more reliable results.^15^ However, acidification of samples for preservation, involving handling of hydrochloric acid (HCl), might be difficult for owners to perform at home. Also, when sampling at home, time from collection to freezing or analysis of the sample at the clinic or laboratory might vary, potentially affecting results.

Analyses of concentrations of catecholamines and metanephrines in canine urine have been performed by high-performance liquid chromatography (HPLC),^4,15,16,22,25^ a method requiring expensive equipment that is not generally available. An ELISA has been validated and found to be simple, rapid, and accurate for detection of NME and ME in human urine, with highly significant correlations to HPLC results.^29^ We evaluated a human ELISA for analysis of metanephrines in feline urine, and found acceptable performance for analysis of NME, except for dilution recovery.^26^

Our aim was to verify a urine ELISA for analysis of the metanephrines NME and ME in canine urine, and to investigate potential effects of acidification, room temperature storage time, sampling time (morning or midday), and time in a clinic environment on concentrations of urine metanephrines.

## Materials and methods

### Animals and study design

We performed our study at the Swedish University of Agricultural Sciences (SLU; Uppsala, Sweden), using urine from privately owned dogs. The study was approved by the Uppsala Ethical Committee, Sweden (approval 5.8.18-18808/2017) and follows the American Society for Veterinary Clinical Pathology quality assurance guidelines for urinalysis in veterinary laboratories.^9^ Written consent was obtained from all dog owners.

Our study consisted of 3 parts: 1) verification of a human commercial reverse competitive urine ELISA kit for analysis of the metabolites NME and ME in canine urine. Based on results of the assay verification, one of the metabolites was selected for further investigation of 2) the stability of the metabolite at room temperature, and the effect of acidification on concentrations of the metabolite, and 3) the effect of sampling time (morning vs. midday) and sampling day (ordinary day vs. day spent in a clinic environment undergoing repeated blood sampling), on concentrations of the metabolite in canine urine.

Healthy dogs were recruited from staff and students at SLU. During the study, the dogs also participated in another study on the influence of different sources of carbohydrates on metabolism. Owners were interviewed concerning the health status of their dogs, and all dogs underwent routine physical examination. For the health examination, a voided urine sample from each dog was obtained. A urinalysis, including protein:creatinine ratio, was performed at the Clinical Chemistry Laboratory of the University Animal Hospital at SLU. Urine protein concentrations were analyzed using an automated multianalyzer (Architect c4000; Abbott). The analyses were performed by a quantitative turbidimetric method with benzethonium chloride and the manufacturer’s reagents.^23,31^ Intra-assay CV was <2% and inter-assay CV was <5% for protein. Urine creatinine concentrations were also analyzed using the automated multianalyzer (Architect c4000). An enzymatic method validated for creatinine in urine and serum was used with the manufacturer’s reagents.^17^ The intra- and inter-assay CV was <2% for creatinine.

To be included in the study, dogs had to be clinically healthy based on history and physical examination (American Society of Anesthesiologists: ASA score 1),^1^ and results of the urinalysis had to be within normal variation, as assessed by urine specific gravity (SG), pH, protein:creatinine ratio, and results of a dipstick chemistry test. Dogs had to be >1 y old and were not permitted to receive antibiotics or any medical hormonal treatment (such as corticosteroids) in the 3 mo preceding the study.

### Sample collection

The owners were informed verbally of how to perform the urine collection and instructed to practice urine collection at home.^13,15^ The samples taken were a total collection at each voiding. In addition to the urine sample for the health examination, repeated voided urine samples were collected by owners throughout the study for inclusion in the different parts of our study. On each occasion, the urine sample was taken to the laboratory and urine SG was measured using a digital refractometer (Pocket refractometer; Atago). The pH was measured by pH paper with a pH scale 0–14 and increments of 1 (MColorpHast; Merck); urine was checked by a dipstick chemistry test, including assessment of leukocytes, nitrite, protein, hemoglobin, ketones, and glucose (Multistix 8SG, Siemens; Table 1).

**Table 1.** Number, urine specific gravity (SG), and pH of urine samples used in our study of catecholamine metabolites.

	Samples (*n*)	Urine SG	Urine pH
Verification	47	1.043 (1.032–1.049)	7 (6–7)
Stability	16	1.051 (1.041–1.053)	6.5 (6–7)
Sampling time and day	116	1.045 (1.035–1.051)	6.5 (6–7)

Values are given as median (interquartile range). Urine SG and pH are for non-acidified samples.

At all urine collections throughout the study, owners were asked to rate the degree of stress they perceived that their dogs experienced during collection, on a scale of 1–4 (1 = none, dog unaffected by urine collection, normal behavior; 2 = mild, dog mildly stressed, but urine collection possible with minor effort; 3 = moderate, dog moderately stressed by the procedure, but urine collection possible with certain effort; 4 = severe, dog severely stressed by the procedure, making urine collection very difficult or impossible). Urine collection was performed between August and December 2019, samples were stored in 2-mL cryotubes (Sarstedt) at −80°C, and analysis of samples was performed between October 2019 and March 2020. The maximum storage time was 6 mo. Aliquots of one urine sample from one dog were used as the canine urine control in all ELISA analyses.

#### Verification study

For the verification study, owners were provided with a plain tube for urine collection and a tube containing 100 µL of 3.2 M HCl. The latter was filled with 2 mL of urine by the owners (marked by a line on the tube). Upon receipt of samples, the pH was checked by pH paper, and if >3 in the acidified sample, additional HCl was added until a pH of 2–3 was achieved. All samples were aliquoted and frozen at −80℃.

#### Metabolite stability study

For evaluation of the effect of time left at room temperature and acidification on concentrations of the metabolite, 16 urine samples were included (Table 1). In the laboratory, each sample was divided into 2 tubes with 2 mL of urine in each tube. Tube 1 was untreated (non-acidified sample) and tube 2 was acidified by adding 100 µL of 3.2 M HCl to the tube. All samples were kept at room temperature (18–23℃). At 0, 2, 4, and 24 h post-collection, aliquots of 0.5 mL of urine were taken from tubes 1 and 2, respectively, transferred to cryotubes, and frozen at −80℃.

#### Effect of sampling time and time spent in the clinic environment

For this part of our study, urine samples were collected by owners in the morning and at midday, on 4 different days. All owners had practiced urine collection at least once before participating in this part of the study. Day 0 was defined as an ordinary day, during which the dog spent the day in its usual environment (at home or accompanying the owner to work). On days 1–3, the morning sample was collected during the same circumstances as day 0; the midday sample was collected after spending ~4 h at the clinic in the feed trial, undergoing repeated blood sampling by an intravenous catheter. Because the dogs participated concomitantly in the feed trial, a potential effect of carbohydrate source on urine concentrations of metanephrines was tested statistically, but had no significant effect (*p* > 0.05).

Day 0 and the first clinic day were performed ~2 wk apart. Clinic day 2 was performed 4–6 wk after clinic day 1, followed by clinic day 3 another 4–6 wk later. All morning samples were collected between 05:30 and 09:00. Midday samples were collected between 11:00 and 13:00 on the ordinary day (day 0), and between 12:30 and 14:30 on clinic days (days 1–3; Table 2).

**Table 2.** Number of urine samples, sampling time of day, time from collection to freezing, and normetanephrine (NME):creatinine ratio in the sub-study evaluating the effect of sampling time and day on NME:creatinine ratio in canine urine.

	Ordinary day (day 0)		Clinic days (days 1–3)	
	Morning	Midday	Morning	Midday
No. of samples	12	12	46	46
Sampling time	07:47 (07:27–08:40)	12:02 (11:36–12:30)	07:15 (06:40–07:47)	13:10 (13:00–13:31)
Time from collection to freezing (min)	30 (25–48)	27 (23–35)	85 (72–135)	51 (35–56)
NME:creatinine ratio (ng/mg)	83 ± 9	96 ± 9	86 ± 7	105 ± 7

Sampling time and time from collection to freezing are medians (interquartile ranges), and NME:creatinine ratios are least square means ± SE.

### Analysis of urine metanephrines

The ELISA analyses were performed according to the manufacturer’s instructions with hydrolysis and acylation of samples before analysis, and with shaking at 600 rpm (Microplate shaker; VWR) during incubations at room temperature. The absorbance was read at 450 nm (Multiskan EX 355 microplate reader; Thermo Fisher). All ELISA analyses were performed by the same researcher (A. Svensson), who was anonymized to dog and sample ID. The reverse competitive human ELISA kit used in our study is available as a combination package for NME and ME (2-Met urine ELISA, BA E-8600; ImmuSmol).

#### Verification study

Verification analyses were performed on acidified samples according to the manufacturer’s instructions, using 5 kits with the same lot number of the combined 2-Met urine ELISA kit. Each urine sample was analyzed in triplicate. The included ready-to-use standards were used. The standard concentrations were 0, 30, 90, 300, 900, and 3,000 ng/mL for NME. The 3,000 ng/mL standard was only used in the first 2 runs and excluded in the following runs because all samples had a concentration <900 ng/mL. For the ME assay, the concentrations of the standards were 0, 20, 60, 200, and 600. As for NME, the 2,000 ng/mL standard was omitted for the same reason after the first 2 runs.

The canine urine control and the 2 human controls provided by the manufacturer were included on each ELISA plate and used to determine inter-assay CVs. Intra-assay CVs were calculated based on triplicate results from all urine samples.^14^ Intra-assay CV was also determined for the canine urine control added in nonuplicate (*n* = 9) within the same plate. All analysis results were included. A CV of ≤12% was considered acceptable because a reasonable number of samples with concentrations within the measuring range fell within this criterion.^6,26^

For determination of dilution recovery, 1 canine urine sample with relative high concentration as well as the high control included in the kit were diluted with standard A (calibrator 0, included in the kit) to 90, 80, 70, 60, 50, 40, 30, 20, 10, 8.3, 6.7, and 5.0%. Diluent for sample dilutions was not specified in the manufacturer’s instruction. For spike-recovery, the canine urine control was spiked 1:1 with the low kit control and the high kit control, respectively. Spike-recovery of 80–120% was considered acceptable.

#### Stability and effect of sampling time and day

Based on results from the verification study, the NME part of the assay, available as a separate kit (Normetanephrine urine ELISA, BA E-8500; ImmuSmol), was used for further analyses. Five kits were used for the stability study, and another 5 kits for the study on sampling time and day. All kits had the same lot number, as for NME in the verification study, and all samples were analyzed in duplicate. Samples with CV >12% were re-analyzed.^26^ All samples had a mean CV <12% upon re-analysis. These mean CVs were accepted and included in the calculations. The canine urine control, as well as the 2 human controls used in the verification, were included as described previously to determine inter-assay CV values. Based on results from the stability study, the analyses on sampling time and day were performed on untreated (non-acidified) urine.

### Statistical methods

The statistical analyses were performed in SAS 9.4 (SAS Institute). Concentrations of NME and ME are presented as least square means (LSmeans) ± SE if nothing else is stated. CV data are presented as mean and total range. Given the non-normal distribution, data on dog characteristics, time intervals, urine SG, and pH are given as median (interquartile range, IQR). Concentrations of acidified samples were corrected for the dilution effect of HCl by 5.0%, as an average dilution of all samples. To correct for urine flow rate when comparing NME concentration between sampling times and days, urine NME:creatinine ratios were calculated; the ratios are given in ng/mg.

Analyses of stability, as well as effect of sampling time and day, were performed using a linear mixed model. For assessment of stability, evaluation of effects of acidification (addition of HCl yes/no) and time at room temperature from collection to freezing (0, 2, 4, or 24 h) on urine NME concentration was performed using the following model:

$$Y_{ijk}=\;\mu \;+a_{i}+\;\beta_{j}+\;\gamma_{k}+\;{\left(\beta \gamma \right)}_{jk}+e_{ijk}$$

where *Y_ijk_* is the observation, µ the mean value, *a_i_* the random effect of the individual dog, β_*j*_ the effect of HCl addition, γ_*k*_ the effect of time at room temperature, (βγ)_*jk*_ the effect of interaction between HCl treatment and time at room temperature, and *e_ijk_* the residuals (i.e., the remaining unexplained variation). Individual*time were set as repeated measurements (with the * indicating an interaction between the 2 parameters).

The effects of sampling day (0 = ordinary day, or 1, 2, 3 = d spent at clinic) and time of day (morning or midday) on urine NME:creatinine ratio were analyzed using a similar model, but where β_*j*_ is instead the effect of sampling day, γ_*k*_ the effect of time of day, and (βγ)_*jk*_ the effect of interaction between sampling day and time of the day.

A possible effect of diet on the urine NME:creatinine ratio was tested using a linear mixed model including the fixed effects of day, time of day, and diet as well as the random effect of the individual dog.

The assumptions underlying the models were checked by preparing diagnostic plots. No apparent deviations from normality or homoscedasticity could be detected. Statistical significance was set at *p* ≤ 0.05. Post-hoc comparisons were adjusted by the Tukey method.

## Results

Of 20 dogs examined, 3 were excluded (1 because of glucosuria, 1 because of proteinuria, and 1 because of severe stress during attempted urine collection [stress score 4], making collection impossible). In remaining dogs, the physical examination and urinalysis were within normal variation (urine sample examined at health examination; median SG 1.041 [IQR: 1.026–1.047], median pH 6.0 [IQR: 6.0–6.5], and urine protein:creatinine ratio ≤0.3 in all dogs). Thus, 17 dogs were included, consisting of 5 mixed breeds, 2 Lagotto Romagnolo, and 1 each of another 10 breeds. The median age was 5.0 y (IQR: 3.2–8.1 y), and median body weight was 15.6 kg (IQR: 10.6–26.4 kg). Eleven of the dogs were females (7 intact, 4 spayed), and 6 were males (1 intact, 5 castrated).

### Verification study

For the acidified samples used in the verification study, the median total HCl volume added to the 2 mL of urine was 100 µL (IQR: 100–120 µL), with a maximum of 140 µL. The median dilution of the samples was 5.0% (IQR: 5.0–6.2%). Median time from collection to acidification was 2 min (IQR: 0–5 min). Median time from collection to freezing was 45 min (IQR: 25–76 min). Dogs were assessed to be unstressed (score 1) by owners at 91% of collections, mildly stressed (score 2) at 7% of collections, and moderately stressed (score 3) at 2% of collections.

#### Normetanephrine

The mean intra-assay CV for urine NME concentration of the canine samples in the 5 verification plates was 6.0% (range: 4.7–6.7%). Eight of the samples had a CV >12%. The intra-assay CV for the canine urine control sample within one plate was 4.3% (concentration: 143 ng/mL; *n* = 9). The inter-assay CVs for the low control sample (mean concentration verification plates: 185 ng/mL), the high control sample (mean concentration verification plates: 575 ng/mL), and the canine urine control sample (mean concentration verification plates: 145 ng/mL) were 3.3%, 3.8%, and 12.0%, respectively. The coefficients of correlation (*R*^2^) for all calibration curves were >0.995.

**Figure 1.**
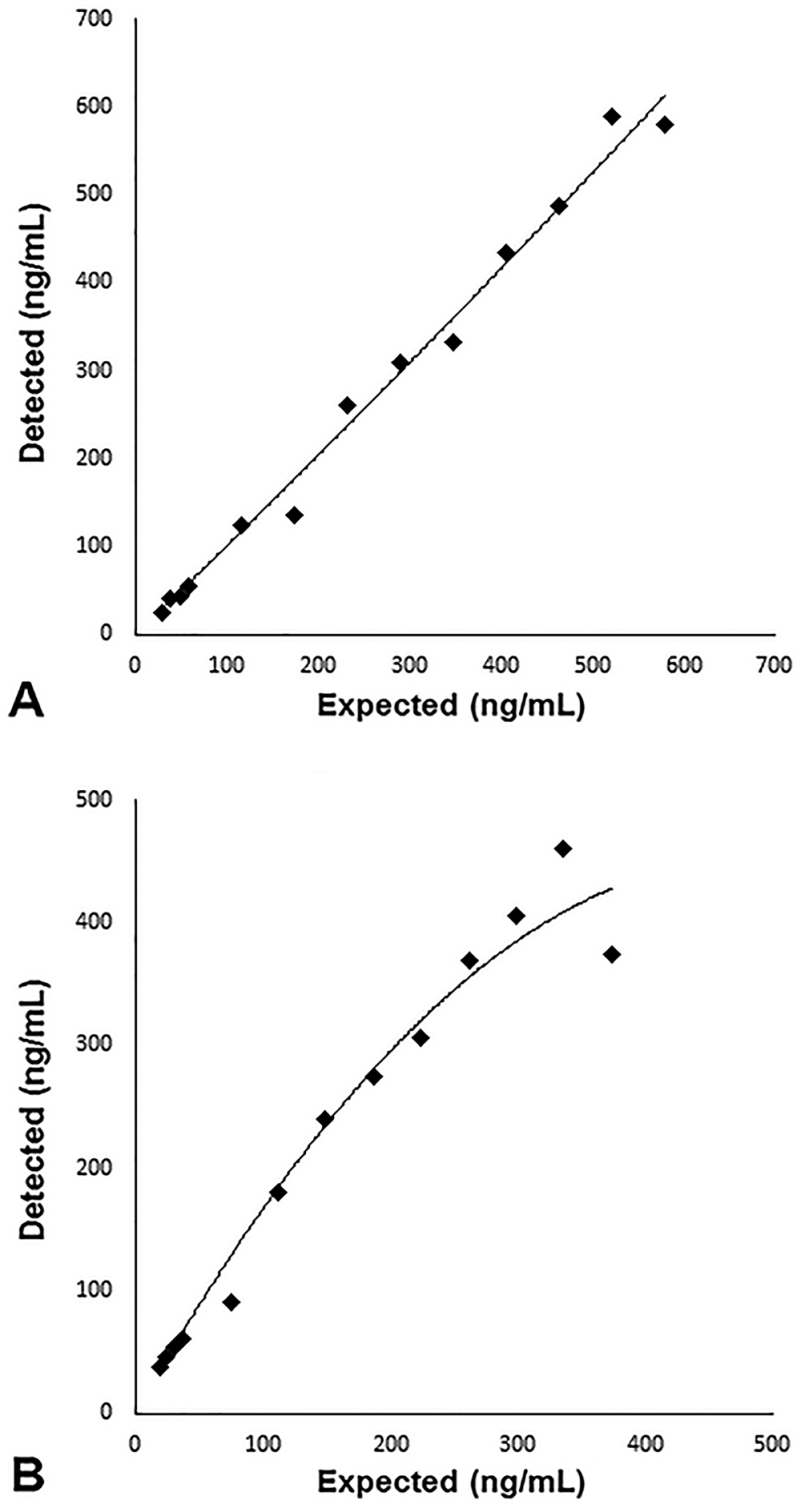
Dilution recovery for normetanephrine in the verification study: **A.** high kit control; **B.** canine sample. A polynomial trend line was applied to each graph.

Spike-recovery of urine NME was 90% when the canine urine control sample was spiked with the low control sample (179 ng/mL), and 101% when spiked with the high control sample (542 ng/mL). Dilution recovery of the high control sample was 78–113%. Dilution recovery of the canine sample was stable, but high, at the first 4 dilutions (90, 80, 70, and 60%, respectively) at 136–141%, and thereafter was 147–198% for the higher dilutions (50–5%; Fig. 1).

#### Metanephrine

The mean intra-assay CV for urine ME concentration of the canine samples in the 5 verification plates was 6.5% (range: 3.7–10.0%). Eleven of the samples had a CV >12%. The intra-assay CV for the canine urine control sample within one plate was 9.0% (concentration: 62.8 ng/mL; *n* = 9). The inter-assay CVs for the low control sample (mean concentration: 126 ng/mL), the high control sample (mean concentration: 384 ng/mL), and the canine urine control sample (mean concentration: 55.5 ng/mL) were 12.7%, 7.2%, and 22.5%, respectively. The *R*^2^ for all calibration curves was >0.996.

Spike-recovery of urine ME was 85% when the canine urine control sample was spiked with the low control sample (133 ng/mL), and 89% when spiked with the high control sample (378 ng/mL). Dilution recovery of the high control sample was 70–115%. Dilution recovery for the canine sample was 108–120% down to 70% dilution, thereafter recoveries were 130–330%.

### Stability study

For time 0 of the stability study, median time from collection to acidification was 5 min (IQR: 0–5 min). Median time from collection to freezing was 15 min (IQR: 15–15 min). Dogs were assessed to be unstressed (score 1) by owners at 93% of collections, and mildly stressed (score 2) at 7% of collections.

**Figure 2.**
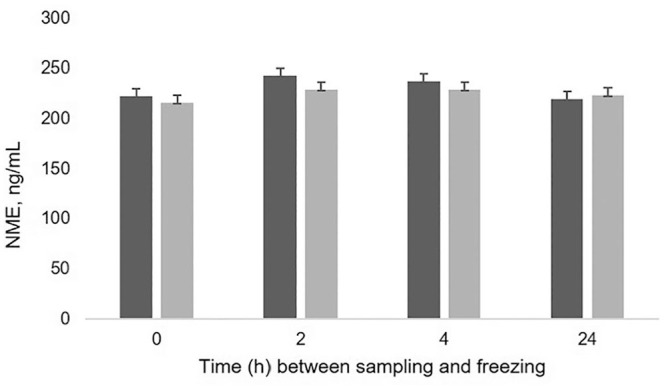
Concentration of normetanephrine (NME) in canine urine samples left at room temperature for 0, 2, 4, or 24 h before freezing. Concentrations are shown as least square means ± SEs; no significant differences, *p* > 0.05. Dark bars = acidified samples (addition of 5% of 3.2 M HCl); light bars = non-acidified samples.

There was no effect of acidification on NME concentration (LSmean ± SE, 230 ± 4 ng/mL with HCl vs. 224 ± 4 ng/mL without HCl, *p* > 0.05; Fig. 2). There was no effect of time at room temperature on NME concentration (*p* = 0.058), nor any effect of interaction between acidification and time at room temperature (*p* > 0.05).

### Effects of sampling time and day

The median time from collection of morning sample to collection of midday sample was 3:57 h (IQR: 3:22–4:40 h) on the ordinary day, and 6:10 h (IQR: 5:30–6:30 h) on days spent at the clinic. Dogs were assessed to be unstressed (score 1) by owners at 97% of collections, and mildly stressed (score 2) at 3% of collections. Hence, no dog was assessed to be moderately (score 3) or severely (score 4) stressed at urine collection.

**Figure 3.**
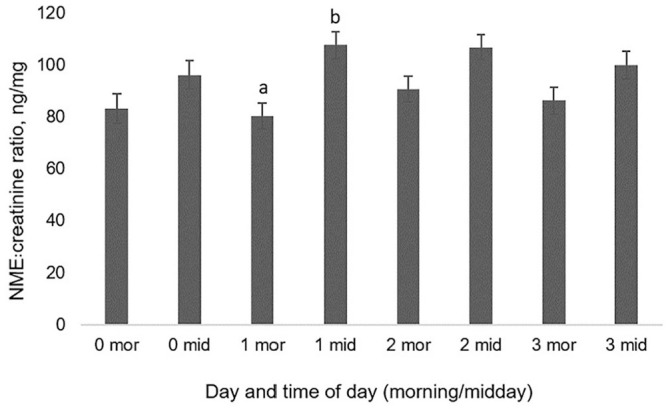
Normetanephrine (NME):creatinine ratio in urine samples from dogs collected twice/day (morning = mor; midday = mid) on an ordinary day (day 0) and during 3 d spent at a clinic (days 1–3). Ratios are shown as least square means ± SEs. Different superscript letters indicate a significant difference between times within day (*p* ≤ 0.05).

We found no difference in the NME:creatinine ratios either within morning samples or within midday samples among the 4 sampling days (*p* > 0.05). The NME:creatinine ratio did not differ between morning and midday on the ordinary day (day 0), or on days 2 and 3 spent at the clinic, but was higher in the midday sample compared to the morning sample on day 1 spent at the clinic (Fig. 3). There was no effect of interaction between day and time of day (morning or midday, *p* > 0.05).

## Discussion

We analyzed the catecholamine metabolites NME and ME using a human urine ELISA. Verification results were acceptable for NME, except for dilution recovery. Based on verification results, NME was selected for the remaining analyses.

Precision for NME was good, with mean intra-assay and inter-assay CVs <12%.^6,26^ Spike-recovery of NME was very good, with values of 90% and 101% when the canine urine control sample was spiked with the low and high human controls included in the kit, respectively. However, spiking analyses were only performed using human controls because no canine control was available. Dilution recovery of the high kit control was acceptable (78–113%). The canine urine sample had stable but high dilution recovery at the first 4 dilutions at 136–141%, and thereafter deteriorated. The high recovery for the 90% dilution indicates a matrix effect in canine urine. Dilutions with shorter intervals might be interesting for future studies. The samples were diluted with standard A (calibrator 0) included in the kit. Dilution recovery might differ using other diluents, but our results indicate that dilution of samples should be avoided.

Compared to NME, the spike-recovery results for ME, at 85% and 89%, were numerically lower. Precision results for ME were acceptable with mean intra-assay and inter-assay CVs ≤12.7% for all samples, with the exception of the higher inter-assay CV for the canine urine control sample. We used a competitive ELISA, which is less sensitive than a sandwich ELISA, hence, higher CVs are expected.^6^ The lower concentrations of ME compared to NME might have contributed to the inferior results of both spike-recovery and dilution recovery for ME compared to NME. Based on these results, dilution of ME at the concentrations that we investigated should be avoided.

Acidification of urine is recommended by the manufacturer of the assay that we used. However, no differences in urine NME concentrations were found between acidified and non-acidified samples. This is good news because samples are acidified commonly with HCl, a strongly acidic solution that can be harmful in contact with the skin or inhaled. Moreover, given that canine urine samples are often collected by the owner of the dog, acidification of samples might be impractical. In addition, we did not detect a significant difference in concentrations at any of the investigated times at room temperature (0, 2, 4, and 24 h), either in acidified or in non-acidified samples. These findings are similar to our study on feline urine, in which NME was shown to be stable at room temperature without acidification up to the maximum tested time of 8.5 h.^26^ In human urine, NME has been shown to be stable at room temperature without acidification for at least 4 d.^28,30^ Thus, our results indicate that canine urine samples can be collected at home and kept at room temperature for up to 24 h without acidification for analysis of NME in the concentration range that we assessed.

We found dogs were assessed by their owners to be unstressed in >90% of collections. A study showed that voided urine collection can initially be perceived as stressful for dogs, and that repeated urine collections at home resulted in a decreasing pattern of the NME:creatinine ratio.^15^ The low apparent stress levels in the dogs of our study might be because dog owners were instructed to practice urine collection at home, and all owners had practiced at least once before taking part in the sub-study evaluating effect of sampling time and day on NME:creatinine ratios. Although slightly numerically higher at midday, there was no significant difference in NME:creatinine ratios between morning and midday sample on the ordinary day (day 0). The lack of diurnal variation in the NME:creatinine ratio is in accordance with other studies in dogs^4^ and humans.^2^ In contrast, several studies show diurnal variation in human excretion of urinary catecholamines^2,18,21^; one study in dogs did not identify circadian variation in urinary catecholamine excretion.^4^

Given that none of the dogs were accustomed to the procedures or to the clinic environment in which these took place, it could be expected that the NME:creatinine ratio in the midday samples would be increased. This was the case during the first day at the clinic (day 1), indicating that the clinic environment and/or the procedures were perceived as stressful for the dogs, in accordance with previous studies.^13,27^ However, although numerically higher, the NME:creatinine ratio was not significantly higher at midday compared to morning on days 2 and 3, potentially indicating accustomization to the procedures and/or environment. All in all, for the dog to be as unstressed as possible, our results, in accordance with other studies, indicate an advantage of sampling at home after practicing urine collection.^13,15,27^ This procedure is simplified by our finding that NME was stable at room temperature up to 24 h without acidification.

Most pheochromocytomas produce primarily norepinephrine, metabolized to NME.^8^ Studies using HPLC have indicated that the urinary NME:creatinine ratio is useful for diagnosis of pheochromocytomas in dogs.^4,15,16,22,25^ Our results suggest that ELISA might be a simple and cost-effective alternative to HPLC for analysis of NME.^29^ However, given that dogs with pheochromocytoma are expected to have substantially higher concentrations of NME than the healthy dogs in our study, validation of the assay at higher concentrations is needed.

The lack of samples with high concentrations of metanephrines is a potential limitation of our study. Dilution recovery of our canine sample had a degree of uncertainty, especially at the highest dilutions for both metabolites. However, NME concentrations of our samples were generally well above those low concentrations, although still below the highest standard. Hence, there was no need for dilution of the samples. In future studies on dogs with pheochromocytoma, in which much higher concentrations will be expected, dilution recovery of canine samples in a higher concentration range needs to be assessed.

For practical reasons, the time from urine collection to freezing was longer on clinic days compared to the ordinary day. However, given that the stability study showed no effect of time at room temperature on NME concentrations, this should not have affected results.
